# Peroxiredoxins and Hypoxia-Inducible Factor-1α in Duodenal Tissue: Emerging Factors in the Pathophysiology of Pediatric Celiac Disease Patients

**DOI:** 10.3390/cimb45020114

**Published:** 2023-02-20

**Authors:** Fadime Aydın Köse, Aysun Pabuccuoglu, Miray Karakoyun, Sema Aydogdu

**Affiliations:** 1 Department of Biochemistry, Faculty of Pharmacy, Izmir Katip Celebi University, Izmir 35620, Turkey; 2 Department of Biochemistry, Faculty of Pharmacy, Ege University, Izmir 35040, Turkey; 3 Department of Child Health and Diseases, Faculty of Medicine, Ege University, Izmir 35040, Turkey

**Keywords:** celiac disease small intestine, inflammation, antioxidant, peroxiredoxin, hypoxia-inducible factor-1alpha

## Abstract

Celiac disease (CD) is an autoimmune enteropathy. Peroxiredoxins (PRDXs) are powerful antioxidant enzymes having an important role in significant cellular pathways including cell survival, apoptosis, and inflammation. This study aimed at investigating the expression levels of all PRDX isoforms (1–6) and their possible relationships with a transcription factor, HIF-1α, in the small intestinal tissue samples of pediatric CD patients. The study groups consisted of first-diagnosed CD patients (*n* = 7) and non-CD patients with functional gastrointestinal tract disorders as the controls (*n* = 7). The PRDXs and HIF-1α expression levels were determined by using real-time PCR and Western blotting in duodenal biopsy samples. It was observed that the mRNA and protein expression levels of PRDX 5 were significantly higher in the CD patients, whereas the PRDX 1, -2, and -4 expressions were decreased in each case compared to the control group. No significant differences were detected in the PRDX 3 and PRDX 6 expressions. The expression of HIF-1α was also significantly elevated in CD patients. These findings indicate, for the first time, that PRDXs, particularly PRDX 5, may play a significant role in the pathogenesis of CD. Furthermore, our results suggest that HIF-1α may upregulate PRDX-5 transcription in the duodenal tissue of CD.

## 1. Introduction

Celiac disease (CD) is a lifelong autoimmune disease of the small intestinal tissue that can occur at any age, from early childhood to old age [1,2,3]. The prevalence of CD has progressively increased over the years, so nowadays, it is one of the most prevalent chronic disorders in children [1,3,4]. 

It is defined by altering degrees of small intestinal damage and clinical symptoms arising by gluten intake [5,6]. In individuals, the immune response induced by the gluten protein causes tissue damage in the proximal intestinal mucosa, characterized by villous atrophy and crypt hyperplasia. However, the reaction that has started in the small intestine affects the whole body system and causes other clinical manifestations such as anemia due to iron deficiency, bone and skin diseases, infertility, endocrine disorders, neurological diseases, and even cancer [3,7,8,9]. Diagnosis of CD is difficult as the symptoms vary between patients. Although many studies have been conducted for years to develop new efficient clinical treatments for CD, the only treatment method is a gluten-restricted diet. 

The pathophysiology of CD is complicated and needs to be thoroughly understood due to the development of novel therapeutic strategies. The mechanism of enterocyte injury during the disease process has yet to be fully explained. The most emphasized mechanisms are discussed under immunological and non-immunological reactions [5,10]. 

The immunological reactions and their effects occur following gluten intake. In the small intestine, gluten is broken down into gliadin and glutenin peptides [11]. Gliadin peptide leads to tissue transglutaminase (tTG) overexpression in intestinal tissue [12,13]. The post-translational modifications of gliadin peptides via tTG trigger and enhance the inflammatory reactions. As a result of T-cell activation, cytokines (e.g., TNF-α, IL-15, IL-21, and IL-18) are released, and the nuclear factor kappa B p65 (NF-κB) and mitogen-activated protein kinase signaling pathways are triggered in patients [10,12,14]. These pathways control the expression of proinflammatory cytokines, adhesion molecules, and nitric oxide synthase (NOS) enzymes. As a result of the activation of inducible NOS (iNOS), NO metabolites are produced, which further increase oxidative stress and inflammation [2,15,16]. Furthermore, tumor necrosis factor-alpha (TNF-α) has potent proinflammatory activities and exhibits considerable apoptotic cell death-inducing features [15,16,17]. Under physiological conditions, the cell turnover rate of small intestinal epithelial tissue is high, which is further increased by CD. Previous studies have reported that epithelial cells die predominantly by apoptosis during the disease process [18,19,20]. However, there are conflicting findings in the literature regarding apoptosis in patients with CD-induced intestinal villus atrophy.

On the other hand, in non-immunological reactions of CD, it has been suggested that gluten exerts a direct cytotoxic effect by causing an increase in reactive oxygen and nitrogen species (ROT and RNT), which are involved in the pathogenesis of CD [3,10,19,21]. In recent years, cellular defense mechanisms related to antioxidant molecules have become critical in diseases affecting autoimmune and other systems such as CD [19,20,22,23].

Peroxiredoxins (PRDX, EC 1.11.1.15) are a multifunctional enzyme family consisting of six isoforms, named PRDX 1, PRDX 2, PRDX 3, PRDX 4, PRDX 5, and PRDX 6, which are expressed in mammalian cells [22,24]. The locational distribution of isoforms in the cell is different. PRDX -1, -2, and -6 are predominantly located in the cytosol, PRDX 3 is found in the mitochondria, PRDX 4 is discovered in ER and is secreted to the extracellular matrix, and PRDX 5 is located in the cytosol, mitochondria, and peroxisomes [24].

PRDXs were first described as antioxidant enzymes. Subsequent research has shown that they also act as redox signaling regulators, chaperones, and proinflammatory factors [25,26,27,28,29,30]. Nowadays, PRDXs are thought to be directly or indirectly associated with many vital cellular pathways such as cell survival and proliferation, apoptosis, development of inflammatory response, neurodegeneration, and resistance to chemotherapeutic agents [24,31,32]. Furthermore, it has been suggested that PRDX isoforms may be important therapeutic targets in various types of cancer including glioblastoma, prostate, colon, and lung cancers [32]. PRDXs are also known to have critical functions in inflammation. Indeed, PRDXs can reduce hydrogen peroxide, alkyl hydroperoxides, and peroxynitrite produced during inflammation. Early published literature expressed that PRDXs play a substantial role in the pathophysiology of inflammatory bowel diseases such as Crohn’s disease and ulcerative colitis [33,34]. However, the findings regarding the expression levels, functions, and regulation of PRDXs in celiac disease are limited to only one article [8]. In this study, it has been reported that the expression level of PRDX 4 increases in the duodenal mucosa of CD disease. However, there are no findings regarding the other five isoforms of PRDX.

Another point is that chronic or prolonged intestinal inflammation can result in an oxygen-restricted environment such as in CD [35,36]. Under decreased oxygen concentration, cellular adaptation systems are activated to ensure the survival of cells. The most important of these adaptation systems is regulated by hypoxia-induced factor-1 alpha (HIF-1α), a transcription factor involved in hundreds of genes that adapts the cell to low oxygen levels under hypoxic conditions [36]. HIF-1α shows its functions through translocating into the nucleus, dimerizing with HIF-1β and binding to hypoxia-responsive elements of the HIF-1α target genes. Recent data have also suggested that HIF-1α plays a role in maintaining intestinal epithelial barrier functions [37,38]. Accumulating evidence has also shown that HIF-1 α plays an essential role in cells via interaction with the NF-kB p65 pathway in the pathogenesis of inflammation [17]. In addition, previous research has further reported that HIF-1α expression is increased in the duodenal tissue of CD patients [19,39]. It has been pointed out that activated HIF-1α is involved in CD pathogenesis. 

It has been revealed that HIF-1α is closely related to PRDX in certain pathologies [32,40,41,42,43]. However, there is no knowledge on the link between PRDX and HIF-1α in CD.

Based on all of these findings, we are of the view that PRDXs may play a significant role in the pathogenesis of CD, and the relationship between HIF-lα and PRDX isoforms observed in other inflammatory diseases may also be possible in celiac disease. Based on the foregoing, we focused on determining the expression levels of PRDX isoforms (PRDX 1–6) and HIF-1α in the small intestinal tissues of celiac patients. 

## 2. Materials and Methods

### 2.1. Subjects and Sample Preparation

Duodenal biopsy samples were collected from seven children (two boys/five girls; median age 9 years, range 5–12 years) with newly diagnosed CD and seven controls (four boys/three girls; median age 11 years, range 8–14 years) during upper gastrointestinal endoscopy who attended Ege University Children’s Hospital, Izmir, Turkey between September 2016 and December 2016. 

All children were diagnosed with CD according to the criteria of the European Society for Pediatric Gastroenterology, Hepatology, and Nutrition (ESPGHN) [44]. The histopathological diagnosis of CD was based on typical mucosal lesions with crypt cell hyperplasia and villous atrophy and was classified according to the MARSH classification (MARSH Type 2a, *n* = 1; MARSH Type 3a, *n* = 2, MARSH Type 3b, *n* = 1, MARSH Type 3c, *n* = 3). In children with newly diagnosed CD, the levels of tissue transglutaminase (tTG) (*n* = 7, mean >200 U/mL) and anti-endomysia IgA (>200 IU/mL) antibodies were higher compared to the reference interval (0–10 IU/mL) (Table 1). Controls were patients referred to the hospital due to various gastrointestinal symptoms (fatigue, chronic abdominal pain, diarrhea) whose endoscopic, histopathologic, and serologic findings and an upper gastrointestinal endoscopy was part of their diagnostic procedure. Their duodenal biopsy specimens showed a normal appearance and histology (MARSH 0, *n* = 7). 

Since this study was planned for pediatric patients, a relatively small population of participants (7/7) was involved. This situation constitutes a limitation of our study. It may be beneficial to design studies with a large sample group in order to more clearly represent the results obtained statistically. Another limitation of this study is that it is not ethically appropriate to obtain biopsies from healthy children by endoscopy. Therefore, the control group in our study was not composed of completely healthy pediatric participants.

Written informed consent was obtained from the parents of each participant prior to the procedure, and the study was approved by the Ege University Faculty of Medicine Clinical Research Ethics Committee (15-3.1/51-2015). 

From each patient, four proximal small intestinal biopsy specimens were obtained. Some of them were used for histopathological analysis and others were washed in ice-cold saline and snap frozen and stored at −80 °C until further analysis for real-time PCR and Western blot analysis. 

### 2.2. RNA Isolation, cDNA Preparation, and Real-Time PCR Analysis

RNA was extracted by using the Gene Jet RNA Isolation Kit (Thermo Scientific, Waltham, MA, USA, #K0731), according to the manufacturer’s instructions. The extracted total RNA concentration of the samples was determined at A260/280 nm by a Beckman-Coulter nanovette spectrophotometer. Reverse transcription was performed with a Maxima H Minus First Strand cDNA Synthesis Kit (Thermo Scientific, #K1681) in a Techne gradient thermal cycler using the following conditions: synthesis for 30 min 65 °C and reverse transcriptase inactivation for 5 min at 85 °C, 40 cycles. The double-stranded DNA dye 2X Maxima SYBR Green qPCR Master Mix Kit (Thermo Scientific, #K0252) was used for quantitative real-time reverse transcription polymerase chain reaction (RT-PCR) analysis with a Roche Light Cycler 480^®^. The amplification specificity of the PCR products of HIF-1α and PRDXs (1–6) was confirmed by the melting curve analysis (data not shown). Forward and reverse primers were designed using Primer3 software [45]. The sequences used are represented in Table 2. GAPDH was used as a housekeeping gene in the experiments performed with tissue samples. The relative expressions of the target genes were quantified according to ABI Prism 7700 Sequence Detection System User Bulletin No. 2 (Applied Biosystems, Foster City, CA, USA) and Schmittengen and Livak [46]. Gene expression was calculated using the 2^−∆∆CT^ analysis. The fold changes were shown as the means ± SEMs in three independent experiments with each triplicate.

### 2.3. Protein Extraction and Western Blotting

Proteins were extracted from biopsy specimens using a buffer containing 150 mM NaCl, %1 NP-40, 50 mM Tris-HCl, 1 mM PMSF, 1x protease inhibitor cocktail (Ambresco, #M221), and 1x phosphatase inhibitor cocktail (Santa Cruz, Dallas, TX, USA, #sc45045) (at pH:8.0) for 20 min on ice. After centrifuged at 15,000 rpm for 15 min at 4 °C, the supernatant was collected and the total protein concentration was determined by the Pierce™ BCA Protein Assay Kit (Thermo Scientific, #23225). Then, 40 µg total proteins per sample were separated on 8–15% sodium dodecyl sulfate-polyacrylamide gels and blotted onto polyvinyl difluoride (PVDF) membranes. The membranes were blocked with TBS buffer containing 0.05% Tween 20 and 5% non-fat dry milk and then incubated with PRDX primer antibodies (listed in Table 3), followed by IR dye conjugated Odyssey^®^ Western blotting kit secondary antibodies (LICOR, Lincoln, NE, USA, #926-31081). Protein bans were visualized in two different wavelengths by the LICOR Odyssey system [47]. All levels of proteins listed in Table 3 were quantified by the SuperSignal^®^ West Pico ECL solution (Pierce, Appleton, WI, USA, #34580). Chemiluminescent signals of the protein bands were detected with a Vilber Lourmat Fusion-FX7 imaging system. The quantitation of blots of the protein bands was conducted by densitometric analysis (Adobe Photoshop software).

### 2.4. Statistical Analysis

Statistical analyses were evaluated by using GraphPad Prism 5.0. software (GraphPad Software Inc., San Diego, CA, USA). For the parametric distribution data, the Student’s *t*-test was used; for non-parametric distribution values, the Mann–Whitney U-test was carried out to determine differences between the CD patients and control groups. A *p* value lower than 0.05 was considered statistically significant. Values were expressed as mean ± SEM.

## 3. Results

### 3.1. tTG and Inflammatory Proteins Expressions in Duodenal Biopsy Samples of CD Patients

**Figure 1:**
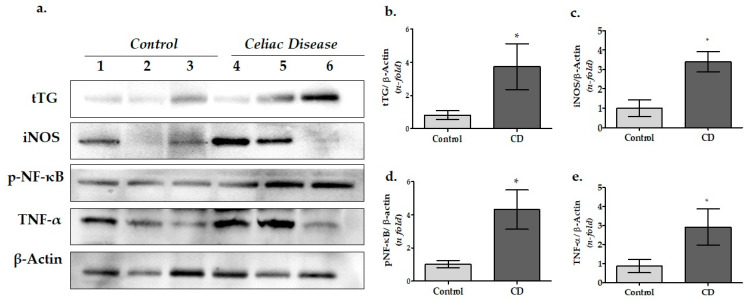
Protein expression levels of tTG and inflammatory proteins in the duodenal tissue of children with CD and the control group. (**a**) Protein expression of tTG, iNOS, p-NF-κB, and TNF-α were determined by WB analysis and quantified by densitometric analysis. Relative densitometric values of the (**b**) tTG protein expression; (**c**) iNOS protein expression; (**d**) p-NF-κB p65 protein expression; and (**e**) TNF-α protein expression levels. The β-actin signal was used to normalize the data, which were then expressed in arbitrary densitometric units. Data were expressed as mean ± SEM (*n* = 3). * *p* < 0.05 versus the control group (CD: celiac disease group).

The protein expression levels of tissue transglutaminase (tTG), inducible nitric oxide synthase (iNOS), tumor necrosis factor- alpha (TNF-α), and phosphorylated nuclear factor kappa B p65 (p-NF-κB p65) were determined in the duodenal biopsy species by Western blotting (WB). It was observed that tTG was significantly overexpressed in pediatric CD patients compared to the control group, as expected (*p* = 0.047) (Figure 1a,b). In addition, the data revealed that the iNOS, p-NF-κB p65, and TNF-α protein levels were significantly higher compared to the control group (*p* = 0.012, *p* = 0.026, and *p* = 0.033, respectively) (Figure 1a,c,e).

### 3.2. Apoptosis-Related Protein Expressions in Duodenal Biopsy Samples of CD Patients

**Figure 2:**
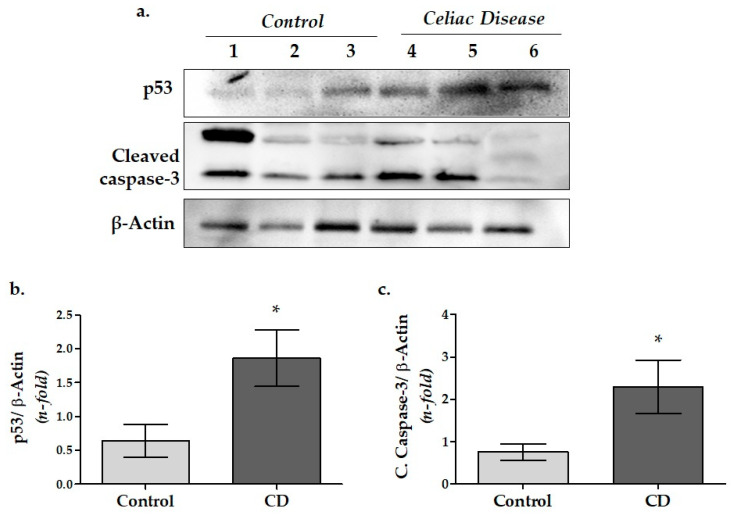
Apoptotic cell death-related cleaved caspase-3 and p53 protein expression levels in the duodenal tissue of pediatric CD patients. (**a**) Protein expression of cleaved caspase-3 and p53 were determined by WB analysis and quantified by densitometric analysis. Relative densitometric values of the (**b**) p53 protein expression and (**c**) cleaved caspase-3 protein expression levels. The β-actin signal was used to normalize the data, which were then expressed in arbitrary densitometric units. Data were expressed as mean ± SEM (*n* = 3). * *p* < 0.05 versus the control group (CD: celiac disease group).

Associated with apoptotic cell death, the p53 and cleaved caspase-3 protein levels were determined in the duodenal mucosa samples of the CD and control group by WB (Figure 2). Results established that cytoplasmic p53 protein expression significantly increased in the CD group upon the control (*p* = 0.033; Figure 2b). In addition, the levels of cleaved caspase-3 in CD were found to be significantly higher than in the control group (*p* = 0.039) (Figure 2c).

### 3.3. HIF-1α Protein and mRNA Expressions in Duodenal Biopsy Samples of CD Patients

**Figure 3:**
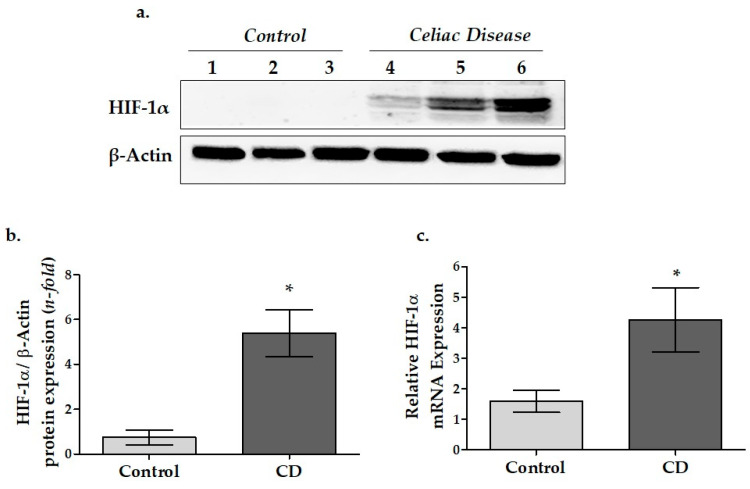
The relative protein and mRNA expression levels of HIF-1α in the duodenal tissue. (**a**) Protein expression of HIF-1α were determined by WB analysis. (**b**) Relative values of HIF-1α were quantified by densitometric analysis. The β-actin signal was used to normalize the data. Data were expressed as the mean ± SEM (*n* = 3). (**c**) The relative mRNA expressions were calculated with the 2^−ΔΔCT^ method and normalized to GAPDH (*n* = 7/7). Data were expressed as the mean ± SEM. * *p* < 0.05 versus the control group (CD: celiac disease group).

The HIF-1α protein levels in the duodenal biopsy samples were determined by Western blot while mRNA expressions were determined using the RT-PCR technique (Figure 3a,b). The HIF-1α protein expression level in CD tissue was observed as approximately 5-fold higher than the control group (*p* = 0.007, Figure 3a). In addition, HIF-1α mRNA expression was found to be significantly increased in the CD patients compared to the controls (4.26 ± 1.05 and 1.59 ± 0.36, respectively) (*p* = 0.002) (Figure 3b).

### 3.4. PRDXs mRNA Expressions in Duodenal Samples of CD Patients

**Figure 4:**
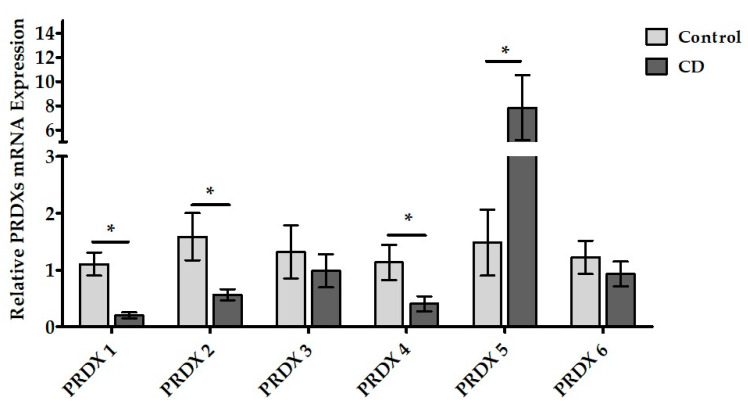
Comparative graph of the relative PRDX (1–6) mRNA expression values. Data were expressed as the mean ± SEM (*n* = 7/7). * *p* < 0.05 versus the control group (CD: celiac disease group).

The mRNA expressions of the PRDX isoforms (PRDX 1–6) were examined using RT-PCR (Table 4 and Figure 4). The data showed that all six PRDX isoforms were transcribed at the mRNA level in the duodenal tissues of the CD patients. It was also observed that some expressions of the PRDX isoforms altered significantly in CD.

Compared to the control group, the PRDX 1, PRDX 2, and PRDX 4 mRNA expressions were significantly decreased in the CD patients (*p* = 0.004, *p* = 0.034, and *p* = 0.011, respectively). In contrast, it was established that PRDX 5 mRNA expression was significantly higher in the CD than in the control group (*p* = 0.037). No significant difference was determined in either the PRDX 3 or PRDX 6 mRNA expression levels between the CD and control groups (*p* = 0.565 and *p* = 0.383, respectively).

### 3.5. PRDXs Protein Expressions in Duodenal Samples of CD Patients

**Figure 5:**
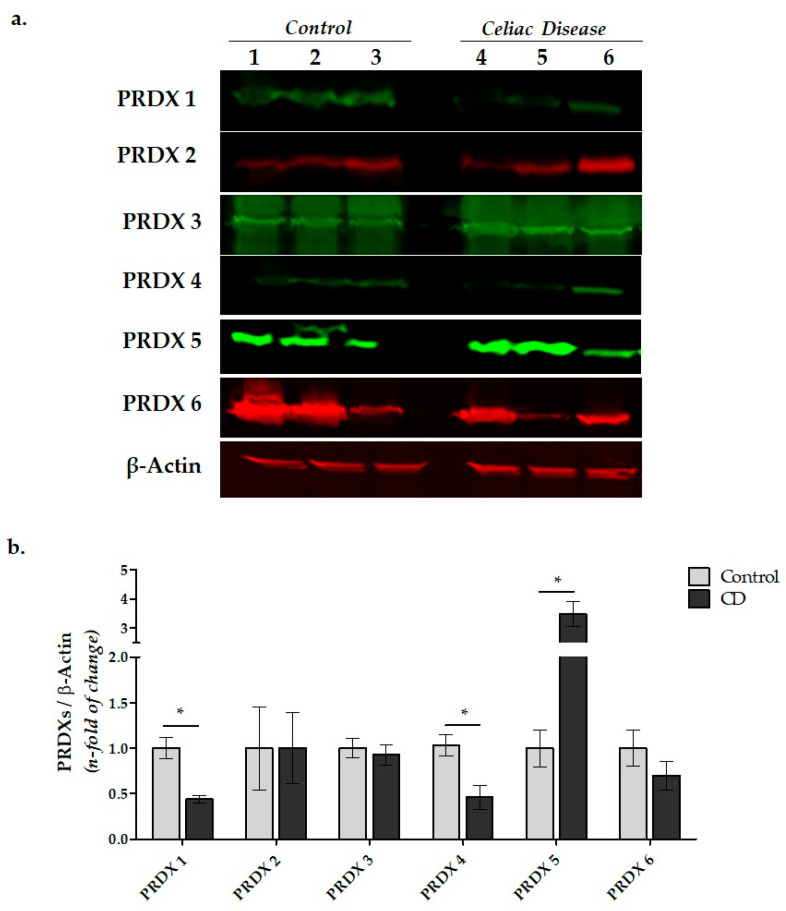
The protein expression levels of the PRDX isoforms in the duodenal biopsy samples. (**a**) Protein expression levels of PRDX -1, -2, -3, -4, -5, and -6 were determined by WB analysis. (**b**) Relative densitometric values of the protein expression levels of the PRDX isoforms. The β-actin signal was used to normalize the data, which were then expressed in arbitrary densitometric units. Data were expressed as the mean ± SEM (*n* = 3). * *p* < 0.05 versus the control group.

The protein levels of the PRDXs in duodenal tissue were analyzed by Western blotting via IR dye conjugated secondary antibodies (Figure 5).

Western blot images revealed that all six enzyme isoforms belonging to the PRDX family were expressed as proteins in the human duodenal biopsy samples. While the protein levels of PRDX 1 and PRDX 4 of the CD group were significantly lower compared to the control, PRDX 5 was found to be significantly higher (*p* = 0.003, *p* = 0.031, and *p* = 0.006, respectively). No significant differences were determined in the PRDX 2, PRDX 3, and PRDX 6 protein expression levels between the two groups (*p* = 0.995, *p* = 0.526, and *p* = 0.234, respectively).

## 4. Discussion

In this study, we aimed to determine the expression patterns of all peroxiredoxin (PRDX) isoforms in the duodenal tissue samples of pediatric celiac patients and to investigate the role of HIF-1α, a transcription factor, in the pathogenesis of the disease.

### 4.1. tTG and Inflammatory Proteins Expression in Duodenal Tissue of Pediatric CD Patients

Initially, tissue transglutaminase (tTG), inducible nitric oxide synthase (iNOS), tumor necrosis factor alpha (TNF-α), and phosphorylated nuclear factor kappa B (p-NFκB), which are associated with CD pathogenesis, were investigated in the samples.

In many previous studies relating to CD, the increase in the tTG in the duodenal tissue has been examined in detail [13,48,49]. Therefore, the tTG level is used as a characteristic marker for CD screening. As expected, high tTG levels in the patient group in our study confirmed that our patient group consisted of celiac patients (Figure 1b).

In addition, our data were consistent with the results of previous studies in reflecting that iNOS, TNF-α, and p-NF-κB protein levels in the patient group were significantly higher than that of the control group (Figure 1c–e). These findings suggest that our celiac patients were in the active phase of the disease and had intense inflammation in their duodenal tissue.

Previous research suggests that gluten peptides cause oxidative stress by producing nitric oxide via the increased activity of iNOS in intestinal epithelial cells. Peterson et al. showed that iNOS expression was induced in the intestinal tissue of CD patients [16]. Daniels et al. investigated the expression of NOS isoforms in duodenal mucosa specimens of CD and iron deficiency anemia [50]. In their study, iNOS mRNA and protein expressions were found to be significantly higher in CD, while neuronal NOS (nNOS) gene expression was not statistically different between groups (Figure 1c). Consequently, iNOS has been found to be a critical downstream mediator of inflammation in various cell types including enterocytes. Our increased iNOS data in CD patients also support this view.

Likewise, Piątek-Guziewicz et al. demonstrated that the expression of TNF-α mRNAs was significantly elevated in adult celiac patients compared to the controls [19,51]. Herein, we found that TNFα was significantly increased in the intestinal tissue of pediatric celiac patients compared to the control group, as shown in Figure 1d.

Meanwhile, the NF-κB signaling pathway is thought to be another regulator of the innate and adaptive immune responses in CD [15,17]. Moreover, it contributes to the regulation of inflammasomes and stimulates the expression of several proinflammatory cytokines and chemokines [14]. According to our findings related to p-NF-κB, different cellular signaling pathways may be activated in the duodenal tissue of celiac patients via inflammation.

### 4.2. Apoptosis-Related Caspase-3 and p53 Protein Expressions in Duodenal Tissue of Pediatric CD Patients

To compare the apoptotic activity in the duodenal tissues of the CD and control group, the cleaved caspase-3 and p53 protein levels were assessed (Figure 2). The results exhibited that the cleaved caspase-3 and p53 levels were significantly elevated in CD than in the control group (Figure 2b,c, respectively). Obtained data indicated that villous atrophy is probably driven by increased apoptosis in CD.

Under physiological circumstances, the turnover rate of the cells in small intestinal epithelial tissue is rapid, which grows further during CD. It has been suggested that epithelial cells predominantly die through apoptosis [18,20,52]. The involvement of the apoptotic pathway in celiac pathogenesis is debatable, and this has been addressed in several papers [20,52,53]. Shalimar et al. reported that CD led to an increased apoptosis in duodenal mucosa that involved both the intrinsic and common pathways [52]. In the same manner, it was reported that pro-apoptotic markers were significantly upregulated, whereas the anti-apoptotic molecules were downregulated in small intestines of the untreated CD patients [20].

Cleaved caspase-3 is the common degradation end-product of apoptosis [18]. Within the present study, we determined that cleaved caspase-3 was significantly increased in the intestinal biopsy samples of untreated CD patients. On the other hand, elevated p53 expression in our CD group implies that activation of the intrinsic apoptotic pathway may occur in enterocytes.

### 4.3. Hypoxia-Inducible Factor (HIF-1α) mRNA and Protein Expressions in Duodenal Tissue of Pediatric CD Patients

Data revealed that HIF-1α protein expression in pediatric CD patients is approximately 5-fold higher compared to the control group (Figure 3a). Additionally, the mRNA expression of HIF-1α was significantly higher in the duodenal mucosa tissue of pediatric CD compared to the controls, as reported in previous studies (Figure 3b).

As in CD, chronic or prolonged intestinal inflammation can result in an oxygen-restricted environment [17,35,54]. HIF-1α is a transcription factor that adapts the cell to low oxygen under hypoxic conditions [35]. Recent data also suggest the importance of HIF-1α in maintaining intestinal epithelial barrier functions [36,37,38,55].

Although HIF-1α activity is primarily regulated by hypoxia, it has been verified that HIF signaling can also be initiated under inflammatory disease [17,56]. Karhausen et al. showed that acute or chronic inflammation in mouse intestinal mucosa epithelial cells led to a hypoxic microenvironment and increased HIF-1α expression [38].

The transcriptional activity of HIF-1α has gained attention as a factor that can regulate intestinal homeostasis, especially in inflammatory disorders [54,57]. Numerous studies have shown that HIF-1α expression and related genes play a role in the pathogenesis of various inflammatory diseases associated with the intestinal system [57,58,59]. Nevertheless, knowledge of HIF-1α in the pathogenesis of CD is limited to only two articles [19,39].

First, Vannay et al. demonstrated that HIF-1α mRNA was overexpressed in the intestinal mucosa tissue of pediatric celiac patients, suggesting HIF-1α signaling involvement in CD pathogenesis [39]. Later, these results were supported by another study indicating that HIF-1α mRNA is overexpressed in the intestinal mucosa of adult CD patients [19].

These findings highlight that HIF-1α and HIF-1α-related genes may play a remarkable role in CD pathogenesis and may be a novel therapeutic target.

### 4.4. Peroxiredoxins mRNA and Protein Expressions in Duodenal Tissue of Pediatric CD Patients

The obtained RT-PCR and immunoblot data revealed for the first time that six peroxiredoxin isoforms were expressed in human duodenal tissue. Furthermore, it has been shown that PRDX 1, PRDX 2, PRDX 4 and PRDX 5 isoforms significantly altered in pediatric celiac patients compared to the control group. These findings support our hypothesis that PRDXs may be involved in the pathogenesis of CD.

In recent years, cellular defense mechanisms related to antioxidant molecules have become critical in diseases affecting autoimmune and other systems such as CD [6]. Reactive oxygen and nitrogen species also facilitate the extensive management of inflammatory processes [10,19,21,60,61,62]. PRDXs have been suggested to represent critical functions in inflammation [30,31,63,64,65,66]. Indeed, PRDXs can reduce hydrogen peroxide, alkyl hydroperoxides, and peroxynitrite produced during inflammation.

Regardless of the CD, studies in the literature reported significant changes in the PRDX levels, especially in the ileum and colon diseases, in studies conducted in the gastrointestinal tract tissues. First, Ishii et al. expressed PRDX 1 in the liver and small intestine tissue in mice [29]. Then, Mo et al. showed that PRDX 6 is also expressed in the murine small intestine [67]. Subsequently, it was stated that the mRNA and protein expression levels of PRDX 6 in the colon tissue of the experimental colitis model created in mice caused a significant decrease [68]. In another study, it was reported that the PRDX 6 expression level was significantly increased in the ileum and colon tissues of Crohn’s disease patients in the case of active inflammation compared to the control group [34]. On the other hand, a study conducted in the small intestine and colon tissue of children with necrotic colitis revealed that only PRDX 1 expression increased in the colon tissue of the patients [69].

The literature is limited to only one article on PRDXs in celiac disease, which reported that the expression level of PRDX 4 in the duodenal mucosa of CD patients was increased [8]. In this study, it was reported that the expression level of PRDX 4 increased in the duodenal mucosa of CD.

In our study, the decrease in PRDX 1, which is defined as the major cytosolic isoform of mammalian cells, in the duodenal tissues of CD patients indicates decreased antioxidant defense and increased inflammation in the duodenal cells (Figure 4 and Figure 5).

Clarifying the relationship between PRDX 1 reduction and duodenal villus atrophy and cryptic hyperplasia may provide valuable results. PRDX 2 mRNA expression was significantly decreased. However, we observed that the PRDX 2 protein levels were found to be similar between the CD patients and control group (Figure 4 and Figure 5). The fact that this transcriptional alteration was not reflected in the protein expression suggests that it may be related to the severity of the disease. Nevertheless, there are studies reporting an increase in PRDX 2 in colorectal cancer [70,71]. PRDX 2 has been reported to promote cell growth and inhibit apoptosis. In addition, it has been demonstrated that PRDX 2 upregulates the Wnt/β-catenin pathway in colon cancer [70,72].

Another remarkable finding of this study is that the mRNA and protein expressions of PRDX 4 were significantly decreased in the patient group (Figure 4 and Figure 5). PRDX 4 is mainly located in ER and is secreted extracellularly. It has been suggested that this isoform can be evaluated as an early serum biomarker in certain diseases such as gastric cancer and cardiovascular diseases [73,74].

Surprisingly, unlike other PRDX isoforms, PRDX 5 was found to be significantly increased in the biopsy samples of CD (Figure 4 and Figure 5). In previous studies, it has been reported that PRDX 5 expression increased in the brain and lung tissues after ischemic brain injury and inflammatory lung disease [32,74]. On the other hand, Kunze et al. investigated the levels of PRDX 5 and various inflammation markers in the plasma samples of patients with cerebral palsy [66]. They found an inverse relationship between the serum inflammatory markers (IL-1, IL-2, IL-6, IL-10, TNF α) and PRDX 5 levels, suggesting that PRDX 5 may be a valuable biomarker in serum.

The present study showed that PRDX 5 mRNA and protein expression is increased in celiac patients (Figure 4 and Figure 5). PRDX 5 can be a more abundant isoform in duodenal mucosa cells compared to others. In addition, the expression patterns of all isoforms revealed that PRDX 5 expression may be controlled by a different mechanism in duodenal tissue. Therefore, it may be important to evaluate the PRDX 5 levels in the serum of celiac patients as a novel biomarker.

In the PRDX 3 and PRDX 6 isoforms, neither the mRNA nor protein expression levels were significantly different compared to the control group (Figure 4 and Figure 5).

All of these findings reveal that peroxiredoxins are proteins involved in the molecular mechanism of CD pathogenesis.

When all of the data in the study were evaluated, it can be said that the increase in the expression of HIF-1α and PRDX 5 in the duodenal tissue of CD patients is a remarkable finding.

## 5. Conclusions

This study showed for the first time that all six isoforms of PRDXs are expressed in human duodenal tissue. The PRDX 5 isoform may have a role in celiac disease pathogenesis and can be evaluated as a potential biomarker in CD patients. Our findings also provide preliminary information on the increased HIF-1α that may positively regulate PRDX 5 expression in the intestinal tissue of CD patients.

Undoubtedly, further studies are needed to reveal the function of PRDXs in CD pathogenesis and to elucidate their relationship with the HIF-1α transcription factor in order to develop new treatment approaches.

## Figures and Tables

**Table 1:** MARSH classification of celiac disease patients.

Patient No.	MARSH Classifications	Serum EMA-IgA ^1^	Serum tTG ^2^ Levels
1	Type 3a	+	>200 IU
2	Type 3b	+++	>200 IU
3	Type 3a	+++	>200 IU
4	Type 2a	+++	>200 IU
5	Type 3c	+++	>200 IU
6	Type 3c	+	>200 IU
7	Type 3c	+++	>200 IU

^1^ EMA-IgA: anti-endomysial IgA antibody; ^2^ tTG: tissue transglutaminase antibody.

**Table 2:** Sequences of the forward and reverse primers used for RT-PCR analysis.

Gene	Forward Primer (5′ → 3′)	Reverse Primer (5′ → 3′)	Base Pair
PRDX-1 (NM_181697.3)	5′tttggtatcagacccgaagc3′	5′agtgaactggaaggcctgaa3′	185 bp
PRDX-2 (NM_005809.6)	5′agatcatcgcgttcagcaac 3′	5′atcctcagacaagcgtctgg3′	182 bp
PRDX-3 (NM_001302272.2)	5′gtcgcagtctcagtggattc3′	5′aacagcacaccgtagtctcg3′	140 bp
PRDX-4 (NM_006406.2)	5′aacagctgtgatcgatggag3′	5′tcaagtctgtcgccaaaagc3′	138 bp
PRDX-5 (NM_012094.5)	5′caagaagggtgtgctgtttg3′	5′taacactcagacaggccacc	134 bp
PRDX-6 (NM_004905.3)	5′atgcctgtgacagctcgtgtg 3′	5′tcttcttcagggatggttgg3′	212 bp
HIF-1α (NM_001530.4)	5′tttccttctcttctccgcgtg 3′	5′ggctgcatctcgagactttt3′	175 bp
GAPDH (NM_002046.7)	5′agccacatcgctcagacac 3′	5′gcccaatacgaccaaatcc3′	65 bp

**Table 3:** Source, dilution ratio, and brand names of the primary antibodies used in the Western blot experiments.

Antibody	Brand	Source	Dilution	Antibody	Brand	Source	Dilution
anti-PRDX 1	Pierce (PA5-29830)	Rabbit	1:3000	anti-iNOS	Pierce (MA3-030)	Mouse	1:250
anti-PRDX 2	Proteintech (60202-1-Ig)	Mouse	1:5000	anti-TNFα	Proteintech (60291-1-Ig)	Mouse	1:100
anti-PRDX 3	Proteintech (10664-1-AP)	Rabbit	1:2000	anti-pNF-κB-p65	Pierce (MA5-15160)	Rabbit	1:500
anti-PRDX 4	Pierce (PA5-34853)	Mouse	1:1000	anti-HIF-1α	Pierce (MA1-16511)	Mouse	1:500
anti-PRDX 5	Proteintech (17724-1-AP)	Rabbit	1:1000	anti-tTG	Proteintech (15100-1-AP)	Rabbit	1:2000
anti-PRDX 6	Pierce (H00009588-M01)	Rabbit	1:1000	Anti-p53	Pierce (MA5-12557)	Mouse	1:100
anti-HIF-1α	Novus Biological (MA1-16511)	Mouse	1:500	Anti-cleaved caspase 3	Cell Signalling (9661)	Rabbit	1:1000
anti-β-Actin	Invitrogen (15G5A11/E2)	Mouse	1:10,000				

**Table 4:** The relative mRNA expression values of the PRDXs (1–6) in the duodenal tissue. The relative mRNA expressions were calculated with the 2^−ΔΔCT^ method and normalized to GAPDH (*n* = 7/7). Data were expressed as mean ± SEM. * *p* < 0.05 versus the control group (CD: celiac disease group).

Gene	Control	CD
PRDX-1	1.105 ± 0.21	0.203 ± 0.05 *
PRDX-2	1.587 ± 0.41	0.566 ± 0.1 *
PRDX-3	1.32 ± 0.47	0.987 ± 0.29
PRDX-4	1.135 ± 0.31	0.407 ± 0.13 *
PRDX-5	1.484 ± 0.58	7.832 ± 2.68 *
PRDX-6	1.226 ± 0.29	0.93 ± 0.22

## Data Availability

Data Availability Statements are available in section “MDPI Research Data Policies” at https://www.mdpi.com/ethics, accessed on 17 February 2023.
